# Flavonoids and Phenolic Acids from Aerial Part of *Ajuga integrifolia* (Buch.-Ham. Ex D. Don): Anti-Shigellosis Activity and In Silico Molecular Docking Studies

**DOI:** 10.3390/molecules28031111

**Published:** 2023-01-22

**Authors:** Fekade Beshah Tessema, Yilma Hunde Gonfa, Tilahun Belayneh Asfaw, Tigist Getachew Tadesse, Mesfin Getachew Tadesse, Archana Bachheti, Devi Prasad Pandey, Saikh M. Wabaidur, Kholood A. Dahlous, Ivan Širić, Pankaj Kumar, Vinod Kumar, Sami Abou Fayssal, Rakesh Kumar Bachheti

**Affiliations:** 1 Department of Industrial Chemistry, Addis Ababa Science and Technology University, Addis Ababa 16417, Ethiopia; 2 Department of Chemistry, Faculty of Natural and Computational Science, Woldia University, Woldia 400, Ethiopia; 3 Department of Chemistry, Faculty of Natural and Computational Science, Ambo University, Ambo 19, Ethiopia; 4 Department of Chemistry, College of Natural and Computational Science, Gondar University, Gondar 196, Ethiopia; 5 Bio and Emerging Technology Institute, Health Biotechnology Directorate, Addis Ababa 5954, Ethiopia; 6 Department of Environment Science, Graphic Era (Deemed to be University), Dehradun 248002, India; 7 Department of Chemistry, Govt Degree College Dehradun Shahar, Suddhowala, Dehradun 248007, India; 8 Department of Chemistry, College of Science, King Saud University, Riyadh 11451, Saudi Arabia; 9 University of Zagreb, Faculty of Agriculture, Svetosimunska 25, Zagreb 10000, Croatia; 10 Agro-Ecology and Pollution Research Laboratory, Department of Zoology and Environmental Science, Gurukula Kangri (Deemed to be University), Haridwar 249404, India; 11 Department of Agronomy, Faculty of Agronomy, University of Forestry, 10 Kliment Ohridski Blvd, 1797 Sofia, Bulgaria; 12 Department of Plant Production, Faculty of Agriculture, Lebanese University, Beirut 1302, Lebanon; 13 Centre of Excellence in Biotechnology and Bioprocess, Addis Ababa Science and Technology University, Addis Ababa 16417, Ethiopia

**Keywords:** *Ajuga integrifolia*, antioxidant activity, binding energies, flavonoids, phenolic acids, shigellosis

## Abstract

Shigellosis is one of the major causes of death in children worldwide. Flavonoids and phenolic acids are expected to demonstrate anti-shigellosis activity and anti-diarrheal properties. The aerial part of *A. integrifolia* is commonly used against diarrhea. This study aimed to identify flavonoids and phenolic acids responsible for this therapeutic purpose. Antioxidant activity, total phenol content, and total flavonoid content were determined. The antibacterial activity of the aerial part against *Shigella* spp. was also tested using the agar well diffusion method. HPLC analysis was performed using UHPLC-DAD for different extracts of the aerial part. Autodock Vina in the PyRx platform was used to screen responsible components. Ciprofloxacin was used as a reference drug. An enzyme taking part in pyrimidine biosynthesis was used as a target protein. Molecular docking results were visualized using Discovery Studio and LigPlot1.4.5 software. Antioxidant activity, total phenol content, and total flavonoid content are more significant for the aerial part of *A. integrifolia*. From HPLC analysis, the presence of the flavonoids, quercetin, myricetin, and rutin and the phenolic acids gallic acid, chlorogenic acid, and syringic acid were identified from the aerial part of *A. integrifolia.* Regarding the antibacterial activity, the aerial part shows considerable activity against *Shigella* spp. Binding energies, RMSD and Ki values, interaction type, and distance are considered to identify the components most likely responsible for the therapeutic effects and observed activity. Antioxidant activity, total phenol content, and total flavonoid content of the aerial part are in line with anti-shigellosis activity. The top five components that are most likely potentially responsible for therapeutic purposes and anti-shigellosis activity are chlorogenic acid, rutin, dihydroquercetin, dihydromyricetin, and kaempferol.

## 1. Introduction

*Ajuga integrifolia* Buch.-Ham. Ex D. Don (Syn: *A. remota*; *A. bracteosa*) belonging to the family Lamiaceae is known by the names *Armagussa*, *Etse Libawit*, *Medhanit*, and *Anamuro* [1]. It is an evergreen, clump-forming flowering species of the genus Ajuga. The more than 300 species of the genus *Ajuga* have many variations [2]. Many biological activities were reported for the *Ajuga* spp., specifically for *A. integrifolia* and its synonyms. These include antioxidant and anti-inflammatory [3], antidiabetic [4], antibacterial, diuretic, stimulant, astringent, rheumatism, febrifuge, blood purification [5], and anticancer effects [6]. These activities are achieved in addition to the biological functions of phytochemicals in protecting plant species against pathogens and herbivores and acting as stress-protecting agents [7].

Diarrhea is a major cause of morbidity and mortality for children in developing countries, including Ethiopia [8,9]. It is among the ten major diseases identified and reported to kill human beings [10]. Shigellosis is a leading cause of dysentery and accounts for 5–10% of diarrheal diseases globally. Shigellosis is characterized by inflammation and ulcer formation on the large intestine, with signs such as fever and stomach pain [11]. It is caused by enteroinvasive *E. coli* (EIEC) and other species of *Shigella*, namely *S. dysenteriae*, *S. flexneri*, *S. boydii,* and *S. sonnei* [12]. *Shigella* spp. are easily differentiated from E. coli, but difficult to differentiate among the species due to similar biochemical traits and modes of invasion [13]. *Shigella* spp. invade the gut-lining epithelium and cause shigellosis [14]. The prevalence of *Shigella* spp. in Ethiopia was reported to be 6.6% and resistant to ampicillin (83.1%), amoxicillin (84.1%), erythromycin (86.5%), and multi-drugs (83.2%). Ciprofloxacin (8.9%) and norfloxacin (8.2%) were reported to have low resistance patterns [15].

*Shigella* spp. infections with increased levels of antimicrobial resistance in children are major public health problems in developing countries [16]. Poor sanitation practices and limited access to clean water are the main reasons for the spread of the disease through the fecal-oral route [17]. *Shigella* spp. is developing resistance to oral antibiotics [18,19,20,21,22]. *Shigella* enters the human body with contaminated food and water as part of its life cycle. It develops an acid-resistant system (glutamate decarboxylase system) which grants the ability to withstand the stomach’s acidic environment [17,23]. Passing through the stomach, the pathogen reaches the intestine and starts invasion. Type III Secretion System (T3SS) mediates *Shigella* invasion and leads to infection [17].

The proliferation of many pathogens, including *Shigella* spp., uses the pyrimidine biosynthesis pathway. *N*-carbamoyl-l-aspartate reversible interconversion to 4,5-dihydroorotate is catalyzed by dihydroorotase (DHO). This makes it a therapeutic target for inhibiting bacterial growth [24]. It is a crucial candidate for evaluating the response to antimicrobial resistance because there is considerable variation between bacterial and mammalian DHOs. Ethnobotanical studies indicate that the aerial part of *A. integrifolia* and its synonyms are used for hypertension [25,26,27,28], antimalarial and insecticidal activities [29,30,31], diuretic activity [32], tonsillitis [33], epilepsy [34], breast cancer [35], wound healing [36], abdominal pain and anthelmintic [37,38,39], and diarrhea [25,40,41,42,43,44]. Individual components have not been identified so far for anti-diarrhea; activity. Flavonoids and polyphenolics are known to have such activity [45,46].

Flavonoids have been discovered in vitro to be potent antibacterial agents against a variety of pathogens, which is not surprising since flavonoids are known to be produced by plants in response to microbial infection. There have been reports of the antibacterial properties of flavonoid-rich plant extracts from various species [47]. Apigenin, galangin, flavone and flavonol glycosides, isoflavones, flavanones, and chalcones are only a few flavonoids that have been proven to have strong antibacterial activity [48]. Rather than focusing on a single site of action, antibacterial flavonoids may have many cellular targets. Their ability to form complexes with proteins by non-specific forces including hydrogen bonds and hydrophobic effects, as well as by forming covalent bonds, is one of their molecular actions. Their capacity to inactivate microbial adhesins, enzymes, cell membrane transport proteins, etc., may therefore be related to their antibacterial activities. Microbial membranes may also be damaged by lipophilic flavonoids [49].

Despite the diversity of natural inhibitors, it is interesting to notice that many of them are phenolic in origin. Many researchers have thought of appropriate skeletons based on the architectures of natural compounds and developed fresh synthetic inhibitors. The previous analysis of tabulated compounds and the inhibitory outcomes point to the flavonol moiety as a potential scaffold, a key structural component, and a starting point for the continued development of novel inhibitors among all the natural and synthesized flavonoid derivatives [50]. Looking for potential inhibitors, in silico molecular docking studies are now becoming part of many pharmacological studies. This strategy produced a conceptual idea for a potential pharmacophore, followed by a targeted chemical synthesis. The application of a ligand-based method and focused chemical synthesis produced novel small synthetic molecules (MW < 500) [51]. After the virtual screening, the short-listed compounds were screened by the SwissADME modeling tool to discard any molecules with undesirable pharmacokinetics and therapeutic qualities. To identify the top binders of the target protein, iterative docking was also used for the drug-like compounds. For the evaluation of the dynamic behavior, stability of the protein–ligand complex, and binding affinity, binding free energy calculations were made, which led to the identification of possible inhibitors [52].

Previous studies indicated that *Shigella* infections were more common in Ethiopia and that they tended to be more resistant to conventional medications such as ampicillin, amoxicillin, and erythromycin [15,21]. A considerable prevalence combined with higher levels of resistance to the currently used drugs brings a demand for a natural-based antibiotic. WHO identified *Shigella* as a marked pathogen against which new drugs need to be formulated and proposed in silico approach to identify drug targets [17]. Considering these, we aimed to justify the anti-shigellosis activity of the aerial part of *A. integrifolia* and identify responsible components for this activity using in silico molecular docking.

## 2. Results

### 2.1. Antioxidant Activity (DPPH Assay)

**Figure 1:**
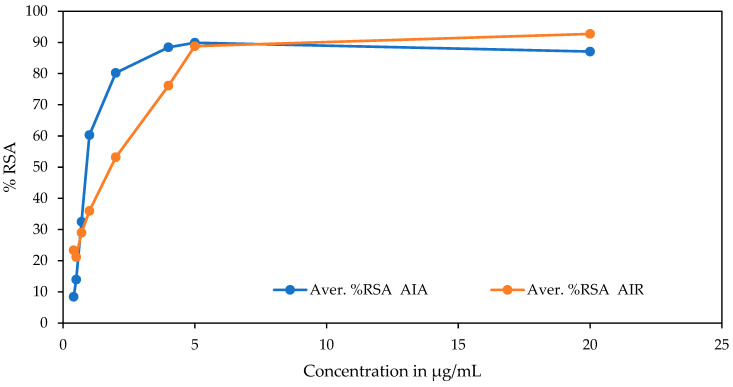
Graph of % free radical scavenging activity of DPPH by the extracts vs. concentration of sample extracts.

The DPPH antioxidant activities of the methanol extracts of the aerial of *A. integrifolia* (AIA) and root part of *A. integrifolia* (AIR) were analyzed using eight concentration points. The antioxidant activity was confirmed first by its color change, which indicated the scavenging activities of the parts of the target medicinal plant. The IC_50_ values of the extracts of AIA and AIR were calculated after being transformed and normalized to the minima (zero) to maxima (plateaus) from log [inhibitor conc.], which are variable responses. The IC_50_ value for the aerial part is significantly lower than the root sample with similar test conditions (Table 1). The antioxidant activity of the aerial part is also much closer to the reference ascorbic acid. The difference in the % of radical scavenging activity of the aerial versus the root sample is observed at lower concentrations (Figure 1). The regression constants (*R*^2^) for the sample extracts and the reference are similar and close to 1.

### 2.2. Total Phenolic and Total Flavonoid Content

Total phenolic content (TPC) and total flavonoid content (TFC) of the extracts of AIA and AIR samples were determined using Folin–Ciocalteu and aluminum chloride methods, respectively. The formation of yellow and blue colors was observed after the addition of the respective reagents for TPC and TFC determinations, respectively. The equation of the standard curve for the determination of TPC was *y* = 0.0023*x* − 0.0693, where *R*^2^ = 0.9981, and for TFC, *y* = 0.002*x* + 0.0399, where *R*^2^ = 0.9971. Both the TPC and TFC of AIA are almost double that of AIR (Table 2).

### 2.3. HPLC Analysis

**Figure 2:**
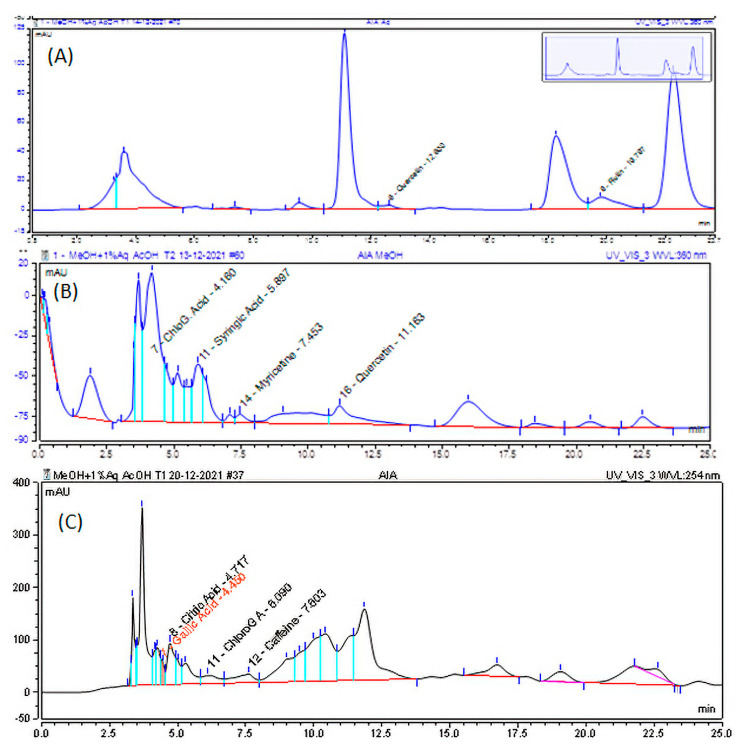
HPLC chromatograms for (**A**) acetone, (**B**) methanol, and (**C**) 75% methanol extract of the aerial part of *A. integrifolia*.

HPLC analysis was carried out using the method mentioned above, and chromatograms were exported for qualitative identification of the presence of phenolic acids and flavonoids. The components from the sample extracts were identified using the reference standards’ peak shape, retention time, and UV-Vis spectra. As shown in Figure 2, the reference peaks were identical and overlaid, leading to the confirmation of the presence of the components. After the identification of the components in the sample extracts, the components were quantified using external calibration standards. From the HPLC chromatogram (Figure 2), gallic acid (RT: 4.450 min), chlorogenic acid (RT: 4.160 min), and syringic acid (RT: 5.897 min) were found in 75% methanol extract; myricetin (RT: 8.117 min) and quercetin (RT: 11.673 min) were found in the methanol extract; and quercetin (RT: 12.600 min) and rutin (RT: 19.797 min) were found in the acetone dip instant extract of aerial part of *A. integrifolia*. As shown in Table 3, the concentration of the phenolic acids and flavonoids ranges from 1.56 to 11.26 mg/100 g for AIR, from 7.97 to 58.23 mg/100 g for methanol extract of AIA (AIA MeOH), from 110 to 220 mg/100 g for acetone extract of AIA (AIA acet), and from 2.07 to 60.13 mg/100 g for 75% methanol extract of AIA. The concentrations are higher for acetone extract as the extraction method is mainly for flavonoids. AIR showed lower concentrations.

### 2.4. Antibacterial Study

The antimicrobial susceptibility test was managed with four strains, including *Shigella* spp. The strains are two Gram-negative and one Gram-positive, chosen based on availability. Methanol extract of AIA showed smaller activity for *E. coli* and a greater zone of inhibition (17.67 ± 1.47) for *Shigella* spp. (Table 4). The ciprofloxacin reference is used following the moderate resistance of *Shigella* spp. for chloramphenicol. AIR does not show any activity for all strains considered.

### 2.5. In silico Molecular Docking Study

Molecular and physicochemical characteristics, including molecular weight (MW), molecular refractivity (MR), count of particular atom kinds, and topological polar surface area (TPSA), can be obtained via the SwissADME online system. Lipophilicity is often described by the partition coefficient between *n*-octanol and water. A soluble molecule facilitates drug development activities, as handling and formulation problems can be managed easily in oral administration, influencing absorption. Lipinski’s Rule of Five and the Bioavailability Radar enable one to decide the drug-likeness of a molecule. Drug-likeness properties describe a molecule’s potential to be an oral drug, or its bioavailability. The resolution of VcDHO (PDB ID: 5vgm) was to be 1.95 Å for the full validation report for the target protein. The overall quality factor retrieved from the PROHECK online server and full validation report was 97.4026%. From the Ramchandran plot, residues in the most favored regions were found to be 90.5% (Supplementary Material S1, Figure S1). No residues were in the disallowed regions. The Q-mean value after preparation was also 0.17 (z-score) (Supplementary Material S1, Figure S2). The resolution, overall quality, and Q-mean values are acceptable for the intended activity.

As shown in Table 5, Lipinski rule violations are tolerable for some molecules, and there were no violations for more than half of the ligands. Only rutin is beyond the tolerable limit of violation. The bioavailability score is above 0.5 for most and 0.1–0.2 for three molecules, including rutin. The synthetic accessibility is also >2.5 for most, including 6.52 for rutin. Medicinal chemistry friendliness is also reflected by lower pain alert and the absence of problematic fragments from Brenk filters [53].

**Figure 3:**
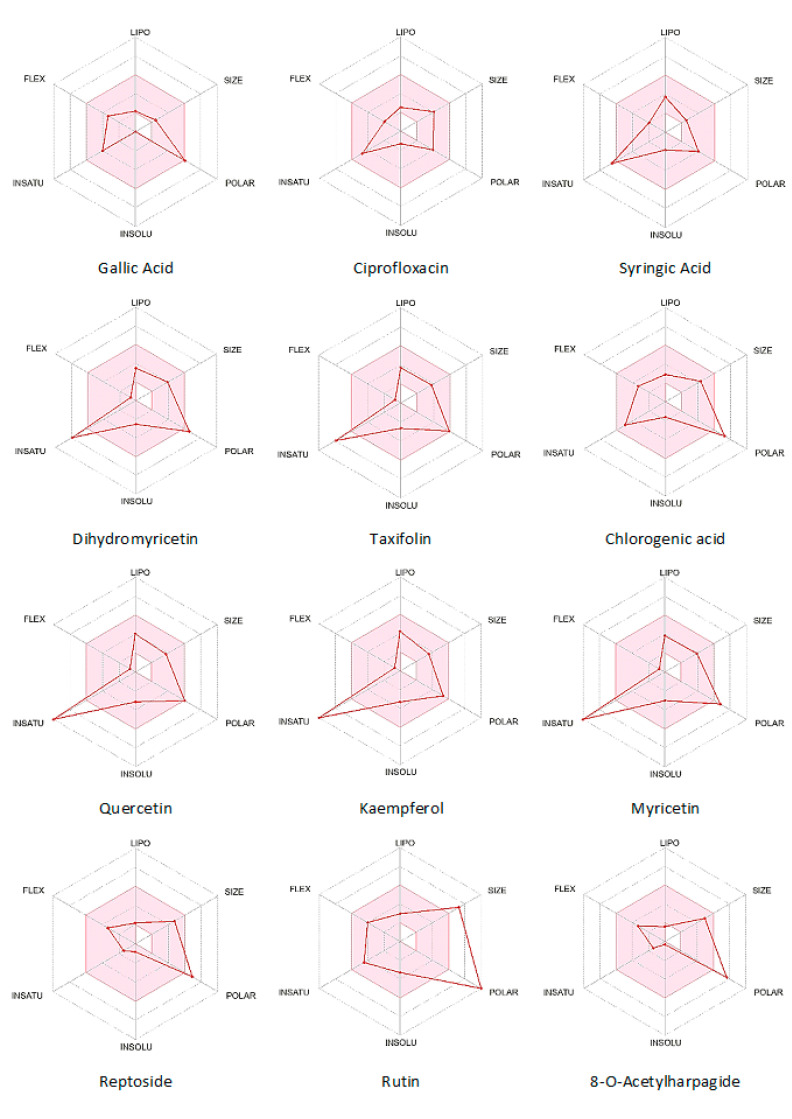
Bioavailability radar for the ligand molecules from SwissADME.

The Bioavailability Radar shown in Figure 3 is used to predict a molecule’s drug-likeness properties. The pink area on the radar represents the acceptable range for each property. The respective values are given as lipophilicity: −0.7 < XLOGP3 < +5.0, size: 150 < MW < 500 g/mol, polarity: 20 < TPSA < 130 Å^2^, solubility: log S < 6, saturation: fraction of sp^3^ hybridized carbons > 0.25, flexibility: number of rotatable bonds < 9. From the Bioavailability Radar (Figure 3), flexibility, lipophilicity, water insolubility, and size are in the acceptable range for most ligands. There exist some anomalies for saturation and polarity in some cases.

The binding energies and interaction study shows a better binding affinity for most as observed to have lower binding energies than the reference drug (Table 6). In addition to the binding energy, the RMSD and Ki of individual ligands are also convincing to consider them for the good inhibitory activity of the target protein. Active site residues identified from the pdbsum online server (http://www.ebi.ac.uk/thornton-srv/databases/cgi-bin/pdbsum/GetPage.pl; accessed 2 December 2022) considering the native ligands and the reference for the PDB data of the target protein [24] are His13, His15, His135, His173, and Asp246.

**Figure 4:**
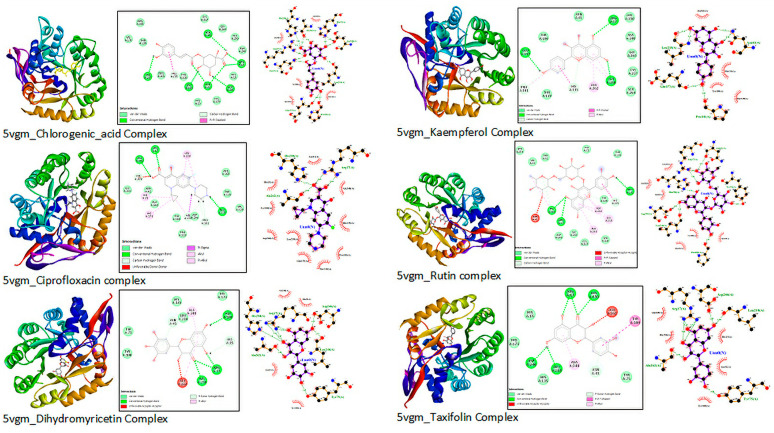
The 3D and 2D views of interactions from Discovery Studio and LigPlot+1.4.5.

The interaction diagrams from Discovery Studio and LigPlot software are similar. The interaction distance considered is less than or equal to 3 nm. Different interactions, including H-bonding, hydrophobic interactions or Van der Waals, Pi-alkyl, Pi-donor H-bond, and Pi-Pi stacked interactions shown in the interaction diagrams from Discovery Studio software in Figure 4.

## 3. Discussion

The antioxidant activities of AIA were observed to be nearly double those of AIR extracts. The IC_50_ value for AIA was found to be lower than the value reported by Lere Keshebo et al. [54]. The computed smaller IC_50_ values of the plant parts considered in our study reveal that this medicinal plant is effective in treating many illnesses [55] due to its high antioxidant activities. This is also shown in various ethnomedicinal studies [25,26,27,28,29,30,31,32,33,34,35,36,37,38,39,40,41,42,43,44] and dictates the presence of polyphenols [45,46] in the extracts more significantly in the aerial part, as demonstrated by Banothu et al. [56]. Similarly, measured TPC and TFC values for AIA were twice those of AIR. This indicates that there is a significant correlation between antioxidant activity and TPC/TFC values [55], and their health benefits are strongly associated. In this study, both TPC and TFC are estimated to be higher, as described in prior investigations [57]. Following higher values of TPC/TFC and antioxidant activity, the antimicrobial activity of AIA against *Shigella* spp. was found to be significant and comparable with other herbs [54] and with ciprofloxacin (Table 4).

The antioxidant activity study result (Table 1) and the total phenol and total flavonoid contents determined (Table 2) indicate the presence of flavonoids and phenolic acids in the aerial part rather than in the root sample. This is in support of the absence of antibacterial activity in the root sample. Phenolics and flavonoids are among the most common components of medicinal plants responsible for antioxidant activities and thus their therapeutic benefits [58]. As per the availability of standards, we checked the presence of flavonoids such as quercetin, myricetin, and rutin and phenolic acids such as chlorogenic acid, syringic acid, and gallic acid. The higher concentration of phenolics in the acetone extract confirmed the presence of flavonoids, as the method [59] is developed for the extraction of flavonoids. HPLC analysis showed that there are also phenolic acid components in the acetone extract in comparable amounts. The identified components from HPLC analysis (Table 3) and others from the literature [60] were considered in the molecular docking study towards screening responsible components for the anti-shigellosis activity observed in the antibacterial study (Table 4) and the therapeutic purpose indicated. The absence of antimicrobial activity for the root part of the plant agrees with the lower concentrations of the phenolics, as shown in Table 3.

Most ligands, except rutin, are known to be Lipinski-compliant and can be considered drug-like molecules. The oral bioavailability test using the Lipinski rule complements the information from the bioavailability radar. Bioavailability and ADMET testing suggest the acceptability of these ligands as a drug [61]. Molecular docking of 5vgm with identified flavonoids and phenolic acids shows that most ligands have better binding affinity than the reference drug (Ciprofloxacin). Binding energy ranges from −8.1 kcal/mole to −5.4 kcal/mol, which is −7.0 kcal/mol for the reference drug. RMSD for the molecular docking for each ligand ranges from 0.1 to 3.4 Å (Table 5). Lower Ki values indicate lower inhibition concentrations for the intended activity and are thus significant. In addition, the lower RMSD values imply consistent docking into the referred active site bounded in the Vina search space indicated above.

This indicates that the environment and parameters set for the molecular docking were reasonably reliable [62]. More robust and relatively stable interactions were observed for most ligands considered. In general, H-bonding interactions dominate over other interactions, including hydrophobic interactions. The role of H-bonding for the overall binding energy is significantly higher [63]. The dihydro-flavonoids, rutin, and chlorogenic acid take the lead in terms of binding affinity. The number of H-bonding interactions is also considerable. Kaempferol and reptoside are comparable to the reference drug in binding energies and RMSD. Myricetin and quercetin also have a suitable binding affinity, slightly lower than the reference drug. Most ligands interact with two or three of the active site residues. Binding interactions with the active site residues also confirm the interaction to be on the right binding site. Common residues such as His250, Arg17, Tyr100, Pro101, and Leu218 appear in most interactions (Table 6 and Figure 4).

Reptoside and 8-*O*-acetylharpagide were considered in the study only for comparison. The antibacterial study shows that the root does not show any significant activity for *Shigella* spp. However, regarding iridoid glycoside composition, both the root and aerial samples were compared when studied using TLC. Iridoid glycosides may not be responsible for the anti-shigellosis activity of the aerial part of *A. integrifolia.* From HPLC analysis, the root sample was found to have an insignificant concentration of flavonoids and phenolic acids. The top five responsible components for anti-shigellosis activity via inhibiting VcDHO are chlorogenic acid, rutin, taxifolin (dihydroquercetin), dihydromyricetin, and kaempferol. The 3-OH and 4-carbonyl functionalities are known to enhance antimicrobial activity. The number of hydroxyl groups is associated with hydrophobicity. Additive hydroxyl groups may lower hydrophobicity, but C3 charges may be increased, which is a clear sign of pharmacological activity [64,65]. The OH groups on the flavonoids are essential for bioactivity, and the change in the position or number of such groups affects biological potency. The plant-derived flavonoid quercetin is a broad-spectrum protein inhibitor [66]. The role of myricetin and quercetin is also not to be undermined following their lower binding energies and lower RMSD and Ki values. The synergic effect will also be considered as there is more than one binding active site for the target proteins and the varied composition of natural products. This demands further study and the activity study of the individual flavonoids and phenolic acids. The observed activity justifies the ethnomedical use of *Ajuga* spp. in traditional medicine. The minimum inhibition concentration (MIC) and minimum bactericidal concentration (MBC) could also be determined to complement the Ki values from the molecular docking study.

## 4. Materials and Methods

### 4.1. Chemicals and Reagents

All chemicals and reagents needed for the extraction, total phenolic content, and total flavonoid content determination and antibacterial activity were AR-grade. Standard references (phenolic acid standards: syringic acid, chlorogenic acid, and gallic acid; flavonoid standards: myricetin, quercetin, rutin, and kaempferol), DPPH, and ascorbic acid for the antioxidant test were all from Sigma (>99.9%, Sigma, Shanghai, China), While for HPLC analysis, all HPLC-grade reagents were used.

### 4.2. Plant Material Collection

The aerial part of *A. integrifolia* was collected from the Addis Ababa Science and Technology University campus and the nearby area of Koye Feche (VRP5 + 3W6, 8.8852 °N 38.8098 °E, elevation: 2840). The plant was identified and an herbarium sample was deposited (voucher number: FB-004/11) in the national herbarium at the College of Science, Addis Ababa University, Ethiopia.

### 4.3. Extraction

Methanol extract of the aerial parts was obtained via Soxhlet extraction using a method by Imoru et al. [67] with slight modifications. Using an ultrasonic probe (SJIA-950W, Sjiolab, Ningbo, China) 75% methanol (aqueous) extraction was performed. The method optimization of UAE followed a method reported by Zakaria et al. [68] with minor modifications. Acetone extract from dip instant extraction, specifically attempted for the extraction of flavonoids, was carried out using the method employed by Mawela [69].

### 4.4. Antioxidant Test—DPPH Assay Calorimetric Method

Modified protocol for the free radical scavenging effect using DPPH assay from Banothu et al. [56] was used, and Soxhlet methanol extracts were tested for antioxidant activity using DPPH as a reagent and ascorbic acid as a standard. We prepared 1000 ppm DPPH and 1000 ppm (mg/L) standard. Ascorbic acid was prepared by dissolving 0.10 g in 100 mL of methanol, and concentrations of 25, 50, 100, 150, 200, 250, 300, 350, 400, 450, and 500 mg/L were prepared for calibration. Absorbance was measured using a Jasco V-770 spectrophotometer (Jasco, Easton, MD, USA), using 1 mm path length in a rectangular cell holder. Sample extracts (0.1 g/mL) of the aerial part and root samples were diluted with CH_3_OH using 5, 10, 25, 50, 100, 150, and 200 dilution factors. Absorbance was measured and recorded in triplicate after 30 min incubation time in a dark place at 517 nm. The proportion of sample to DPPH was 1:3, i.e., 750 mL of DPPH added to 250 mL of sample, which was modified for some cases. DPPH scavenging capacity was computed by using the following formula:

(1) $$\mathrm{Scavenging}\;\mathrm{activity}\;\left(\%\right)=\left(\frac{{\mathrm{Absorbance}}_{\mathrm{control}}-{\mathrm{Absorbance}}_{\mathrm{sample}}\;}{{\mathrm{Absorbance}}_{\mathrm{control}}}\right)\times 100$$

IC_50_ values were computed from the relation log [sample] vs. absorbance (normalized) using Graph pad prism 8 software, as suggested for better EC_50_ estimation [70].

### 4.5. Total Phenolic Content (TPC) Determination: FC Method

The total phenolic content of aerial and root samples of *A. integrifolia* was determined by a colorimetric method Folin–Ciocalteu (FC) assay, as described by McDonald et al. [71] with some modifications. First, 0.4 mL of sample extract and 0.4 mL of FC reagent (10× diluted) were mixed, and then 0.2 mL of 2% Na_2_CO_3_ was added after 5 min. Using a V-770 UV-Vis spectrophotometer, absorbance at 765 nm was measured after the mixture had been incubated at room temperature for 35 min. The control was methanol. A standard solution of gallic acid for the calibration curve was prepared in 1000 ppm (mg/L) and serially diluted to 6.25, 12.5, 25, 50, 100, 150, 200, 250, and 500 mg/L standard solutions. The calibration curve was created using the average absorbance values at the appropriate concentrations from each experiment performed in triplicate. TPC was calculated as the milligrams of gallic acid equivalent (GAE) for each extracted material gram (dry weight).

### 4.6. Total Flavonoid Content (TFC): Aluminum Chloride Method

With slight adjustments, the aluminum chloride colorimetric method reported by Chang et al. [72] was used to measure the extracts’ total flavonoid concentration. Briefly, the mixture of 0.3 mL sample extract, 0.3 mL of 2% AlCl_3_, 0.3 mL 1% sodium nitrite, and 0.3 mL 5% NaOH was mixed. The mixture was incubated at room temperature for a total of 30 min. Methanol was used as a control. Absorbance was measured at λ 314 nm using the spectrophotometer mentioned above. Quercetin prepared at 1000 ppm (mg/L) was used as a standard and calibration concentrations were prepared for TPC determination. TFC was computed as mg of quercetin equivalent (QE) per gram (dry weight) of sample extract.

### 4.7. HPLC (UHPLC-DAD) Analysis

Ultra-high-performance liquid chromatography coupled with a diode array detector (Ultimate-3000 UHPLC-DAD, Thermo Scientific Dionex, Sunnyvale, CA, USA) was used to determine the presence of flavonoids and phenolic acids. The column was in reverse phase with Fortis 5 mm C18 (4.6 × 250 mm column dimension). Methanol-acidified (1% acetic acid) ultra-pure water (60/40, *v/v*) at the flow rate of 0.8 mL/min was used as the mobile phase. Autosamplers and column temperature were set at 25 °C and 35 °C, respectively. Then, 10 μL of the purified sample extracts dissolved in the mobile phase mixture was injected into the column, and UV-Vis detection was attained at 254 nm, 272 nm, 360 nm, and 372 nm. The standard mixture of the phenolic acids syringic acid, chlorogenic acid, and gallic acid, and the flavonoids quercetin, myricetin, and rutin at the concentrations of 2.5, 10, 20, 40, 50 mg/mL were used as the external reference standard mixtures.

### 4.8. Antibacterial Tests

An antimicrobial efficacy study was conducted in the Ethiopian Biotechnology Institute, Ethiopia Microbiology Laboratory.

#### 4.8.1. Test Organisms

*Escherichia coli* (ATCC 25922)*, Pseudomonas aeruginosa* (ATCC 27853)*,* and *Staphylococcus aureus* (ATCC 25923) were obtained from the Ethiopian Public Health Institute. *Shigella* spp. was obtained from clinical isolates selected by a researcher at Bio and Emerging Technology Institute (formerly EBTi), Ethiopia.

#### 4.8.2. Antibacterial Activity

Selected test organisms were subjected to susceptibility tests for each extract using the agar well diffusion method as used by Lulekal et al. [73] with slight modifications. The susceptibility test was performed in triplicate for the methanol extract of the root and aerial part of *A. integrifolia*. Test organisms were swabbed onto sterile Mueller Hinton agar (HIMEDIA, Mumbai, India) plates using sterile cotton swabs. Mueller Hinton was swabbed, the agar was allowed to dry, and the blue points were used to drill 6 evenly spaced holes. Then, 100 μL of one sample extract (with a concentration of 0.1 g/mL) was placed in three holes, while 100 μL of the other extract was placed in the remaining three holes. For the negative and positive controls, equal volumes of sterile distilled water, methanol (diluent), chloramphenicol, and ciprofloxacin suspensions were used. The zones of inhibition (ZOI) (susceptibility or resistance) of the extracts and control for each test organism were then measured with a ruler and reported in millimeters after incubation at 37 °C for 24 h.

### 4.9. In Silico Study

#### 4.9.1. Physicochemical and Pharmacokinetic Properties

The SwissADME web tool (http://www.swissadme.ch; accessed 2 December 2022), with free access to a pool of fast and robust predictive models including built-in methods such as the BOILEDEgg, iLOGP, and Bioavailability Radar, was used to retrieve properties such as physicochemical, pharmacokinetics, and drug-likeness. Medicinal chemistry friendliness was checked using two models: PAINS alert and the Brenk filter models [53]. Easy, efficient input formats and interpretation are made possible through a user-friendly interface.

#### 4.9.2. Molecular Docking: Interaction Study

**Figure 5:**
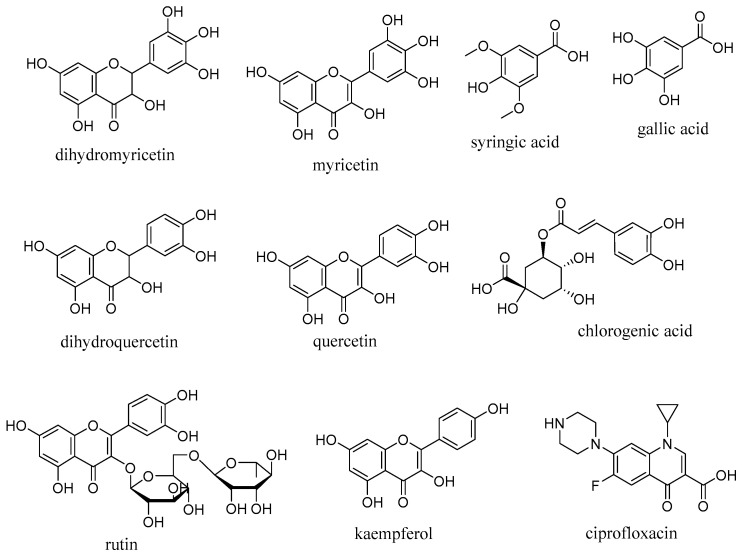
Structures of ligands selected for the in silico study.

For the selection and preparation of target protein and ligand structures, DHO is preferred for its essential role in the proliferation of pathogens and as one of the key enzymes in the aforementioned pathway [24]. Lipowska and coworkers proposed two structures of DHOs, the plague-causing pathogen from Yersinia pestis (YpDHO) (PDB ID: 6CTY), and the causative agent of cholera from *Vibrio cholerae* (VcDHO) (PDB ID: 5VGM). We selected the latter one after checking the crystal structure resolution, total quality factor, and Q-mean values [74] and testing for docking with the reference drug on the respective active site. The ligands used in this study were the flavonoids and phenolic acids identified in the HPLC analysis and from the literature [75]. The structure of these ligands is shown in Figure 5. Ciprofloxacin was used as a reference after its first-line treatment for shigellosis [8,76]. Preparations of the receptor protein and ligands were managed after the structure retrieved from pdb (https://www.rcsb.org/structure/5VGM; accessed 2 December 2022) and PubChem (https://pubchem.ncbi.nlm.nih.gov/; accessed 2 December 2022) online servers using standard procedure [77]. The structures were minimized using USCF Chimera 1.15 software (Resource for Biocomputing, Visualization, and Informatics University of California, San Francisco, CA, USA) and ChemDraw 3D software (Version 12.0, PerkinElmer, Waltham, MA, USA). The prepared structures were saved as pdb files and made ready for molecular docking.

#### 4.9.3. Molecular Docking and Visualization

Prepared structures of the receptor macromolecule and ligand molecules were loaded in PyRx 8.0 software [63] and converted to the respective pdbqt files. Using the information about the residues of the active site, Vina search parameters were set as follows: exhaustiveness = 8; center *x* = 21.5380864417, *y* = 18.4743434105, *z* = 82.1235234094; dimensions *x* = 30.1832504022, *y* = 30.810349529, *z* = 30.3537531811. After completing the Vina, a CSV output file with pose binding energies and respective RMSD values was generated for each ligand on an Excel sheet and output for further interaction study using visualizing software such as PyMol 2.5 (Schrödinger, Mannheim, Germany), Discovery Studio (Dassault Systèmes, Vélizy-Villacoublay, France) [78], and LiPlot+1.4.5 (European Bioinformatics Institute, Cambridge, United Kingdom) [79]. From the visualizing software’s interaction types, their distance and interacting residues were extracted and used for the discussion and conclusions.

## 5. Conclusions

The search for novel and effective therapeutic targets has become necessary due to the rise in organisms’ antimicrobial resistance tendencies. The antioxidant activity of the aerial part of *A. integrifolia* was more significant than the root sample. This indicates the presence of phenolics and flavonoids in the aerial part rather than in the root sample, as was also confirmed for the antibacterial activity. Following the considerable activity of the aerial part against *Shigella* spp., potential anti-shigellosis activity was screened. VcDHO was selected as a drug target for its role in the proliferation of pathogenic bacteria. As responsible components, flavonoids and phenolic acids identified from HPLC analysis and others from the previous study in the aerial sample of *A. integrifolia* were considered. Most likely, chlorogenic acid, rutin, taxifolin (dihydroquercetin), dihydromyricetin, and kaempferol are potentially responsible for the anti-shigellosis activity and therapeutic potential. The synergic effect and specific activity of the compounds identified as potential inhibitors of VcDHO are essential to reaching a more robust conclusion.

## Figures and Tables

**Table 1:** Result summary of IC_50_ and *R*^2^ values.

Sample	Log(inhibitor) vs. Normalized Response—Variable Slope		
	LogIC_50_	IC_50_ (μg/mL)	*R* ^2^
AIA	−0.053 ± 0.01	0.885 ± 0.02	0.991 ± 0.001
AIR	0.318 ± 0.004	2.080 ± 0.017	0.990 ± 0.002
Ascorbic acid	−0.908 ± 0.01	0.124 ± 0.01	0.991 ± 0.02

AIA: *A. integrifolia* aerial part, AIR: *A. integrifolia* root part.

**Table 2:** Total phenol content and total flavonoid content for the sample extracts.

Sample	TPC—Total Phenolic Content (mg/100g)	TFC—Total Flavonoid Content (mg/100g)
AIA	868.98 ± 9.98	742.44 ± 24.47
AIR	475.79 ± 4.35	391.98 ± 6.44

AIA: *A. integrifolia* aerial part, AIR: *A. integrifolia* root part.

**Table 3:** HPLC quantitative result for *A. integrifolia* extracts.

Sample/Extracts	Mass	DF	Analytes	Ret. Time (min)	Amount (mg/L) AV ± SD	Concentration (mg/100 g) AV ± SD
AIA	5.0026	1	Chlorogenic Acid	4.237	23.66 ± 1.6	23.65 ± 1.6
AIA	5.0026	1	Gallic Acid **	4.060	60.16 ± 0	60.13 ± 0
AIA	5.0026	1	Myricetin	8.107	7.85 ± 1.07	7.84 ± 1.07
AIA	5.0026	1	Quercetin	11.967	2.07 ± 0.55	2.07 ± 0.55
AIA	5.0026	1	Syringic Acid	6.093	64.22 ± 5.48	64.18 ± 5.48
AIA	5.0026	1	Rutin	-	-	-
AIA Ac *	0.3	2	Chlorogenic Acid	4.287	6.61 ± 1.63	220.4 ± 54.25
AIA Ac *	0.3	2	Gallic Acid	-	-	-
AIA Ac *	0.3	2	Myricetin **	7.723	3.31 ± 0	110.27 ± 0
AIA Ac *	0.3	2	Quercetin	-	-	-
AIA Ac *	0.3	2	Syringic Acid	5.997	4.94 ± 2.2	164.59 ± 73.36
AIA Ac *	0.3	2	Rutin	-	-	-
AIA MeOH	5.0026	3	Chlorogenic Acid	4.183	19.42 ± 1.24	58.23 ± 3.72
AIA MeOH	5.0026	3	Gallic Acid **	4.030	8.12 ± 0	24.34 ± 0
AIA MeOH	5.0026	3	Myricetin	7.463	3.71 ± 0.72	11.12 ± 2.17
AIA MeOH	5.0026	3	Quercetin	11.873	3.8 ± 1.95	11.4 ± 5.86
AIA MeOH	5.0026	3	Syringic Acid	5.897	9.77 ± 1.97	29.3 ± 5.9
AIA MeOH	5.0026	3	Rutin	-	-	-
AIR	5.008	1	Chlorogenic Acid	4.31	7.98 ± 1.45	7.97 ± 1.45
AIR	5.008	1	Gallic Acid **	3.957	11.28 ± 0	11.26 ± 0
AIR	5.008	1	Myricetin	7.437	2.51 ± 0.91	2.51 ± 0.9
AIR	5.008	1	Quercetin	11.677	1.57 ± 0.56	1.56 ± 0.56
AIR	5.008	1	Syringic Acid	5.997	3.54 ± 1.81	3.53 ± 1.81
AIR	5.008	1	Rutin	-	-	-

* Concentration computed from extract mass. ** Quantitative determinations from a single measurement. DF, dilution factor; -: not applicable.

**Table 4:** Measurement of the zone of inhibition (mm) of test bacteria.

S. No.	*E. coli*	*P. aeruginosa*	*S. aureus*	*Shigella* spp.
AIA	3.33 ± 0.82	0	0	17.67 ± 1.47
AIR	0	0	0	0
Chloramphenicol	-	-	-	10.33 ± 0.82
Ciprofloxacin	-	-	-	20.33 ± 1.08

AIA: *A. integrifolia* aerial part, AIR: *A. integrifolia* root part.

**Table 5:** Physicochemical and pharmacokinetic properties of ligands retrieved from SwissADME (Supplementary Material S2).

Molecule	MW	# Rotatable Bonds	# H-bond Acceptors	# H-bond Donors	MR	TPSA	iLOGP	GI Absorption	BBB Permeant	Pgp Substrate	CYP3A4 Inhibitor	Lipinski # Violations	Bioavailability Score	PAINS # Alerts	Synthetic Accessibility
Gallic Acid	170.12	1	5	4	39.47	97.99	0.21	High	No	No	Yes	0	0.56	1	1.22
Ciprofloxacin	331.34	3	5	2	95.25	74.57	2.24	High	No	Yes	No	0	0.55	0	2.51
Syringic Acid	198.17	3	5	2	48.41	75.99	1.54	High	No	No	No	0	0.56	0	1.7
Dihydromyricetin	320.25	1	8	6	76.78	147.68	0.67	Low	No	No	No	1	0.55	1	3.55
Taxifolin	304.25	1	7	5	74.76	127.45	0.71	High	No	No	No	0	0.55	1	3.51
Chlorogenic Acid	354.31	5	9	6	83.5	164.75	0.87	Low	No	No	No	1	0.11	1	4.16
Quercetin	302.24	1	7	5	78.04	131.36	1.63	High	No	No	Yes	0	0.55	1	3.23
Kaempferol	286.24	1	6	4	76.01	111.13	1.7	High	No	No	Yes	0	0.55	0	3.14
Myricetin	318.24	1	8	6	80.06	151.59	1.08	Low	No	No	Yes	1	0.55	1	3.27
Reptoside	390.38	5	10	5	87.44	155.14	1.92	Low	No	Yes	No	0	0.55	0	5.6
8-*O*-acetylharpagide	406.38	5	11	6	88.6	175.37	1.36	Low	No	No	No	2	0.17	0	5.75
Rutin	610.52	6	16	10	141.38	269.43	0.46	Low	No	Yes	No	3	0.17	1	6.52

#: number.

**Table 6:** Result summary of molecular docking analysis of ligands with VcDHO protein (PDB ID: 5vgm).

Ligand	Binding Energy (kcal/mol)	RMSD (Å)	KI (μM)	Interacting Residues	
				H-Bonding	Others
Chlorogenic acid	−8.1	3.416	1.15	**His15**, Arg17, **Asp246**, Pro101, Asn41, Ala262, Leu218, Glu137, His250	Ala248, **His135**, Thr139, Tyr100, Gly263,
Rutin	−8.1	2.264	1.15	Arg17, His250, Leu218, Tyr75, Pro101, Asn41, **Asp246**	**His135**, Tyr100, Ala262, Thr139, Ala248, Gly263
Taxifolin	−7.4	1.648	3.76	Arg17, **Asp246**, Ala262,Tyr75, Leu218	Asn41, **His15**, Ala248, Tyr100, His250
Dihydromyricetin	−7.3	0.546	4.45	Arg17, Ala262, **Asp246**, Tyr75, His250, Leu218	**His15**, Ala248, Asn41, Tyr100
Kaempferol	−7.2	1.491	5.27	Leu218, Pro101, Asn41, Glu137	**His135**, Tyr100, Thr139, Ala262
Reptoside	−7.1	1.852	6.24	Asn41, **His135**, Tyr75, Ala262, Leu218, **Asp246**	Tyr100, **His15, His173**
Ciprofloxacin	−7	2.267	7.39	Arg17, His250, Ala262	**His15**, Asn41, **His135**, Pro101, Glu137, Thr139, **Asp246**, Leu218, Tyr100, Ala248
Myricetin	−6.9	2.042	8.74	Pro101, His250, Ala262, Glu137, **Asp246**	Tyr100, **His15**, Thr139, Leu218, Ala248, **His135**
Quercetin	−6.6	1.977	14.51	Pro101, His250, Ala262, Glu137, **Asp246**	Tyr100, **His135, His15**, Thr139, Leu218, Ala248
8-*O*-Acetylharpagide	−6.3	1.121	24.07	Asn41, Leu218, Arg17	**His15**, Tyr100, **His135**, Ala262, Ala248, His250
Gallic acid	−6.2	0.509	28.5	**Asp246**, Arg17, Leu218, Asn41, **His135**, His250, **His173**, Ala262	**His15**, Tyr100, Ala248
Syringic acid	−5.4	0.146	109.99	Asn41, **Asp246,** His250, Arg17, Ala262	Tyr100, **His135**, Leu218, Ala248, Gly263, Cys217

## Data Availability

This article and its supplemental information files contain all the data created or analyzed during this investigation.

## Supplementary Materials

The following supporting information can be downloaded at: https://www.mdpi.com/article/10.3390/molecules28031111/s1, Supplementary Material S1: Figure S1: Main Ramachandran plot for 5VGM; Supplementary Material S1: Figure S2 Quality comparison for 5VGM; Supplementary Material S2: SwissADME data for ligands.
