# Supplementary figures and images for: Flavonoids and Phenolic Acids from Aerial Part of Ajuga integrifolia (Buch.-Ham. Ex D. Don): Anti-Shigellosis Activity and In Silico Molecular Docking Studies
Source: Molecules. 2023 Jan 22;28(3):1111. doi: 10.3390/molecules28031111 (PMC9920895; doi:10.3390/molecules28031111)

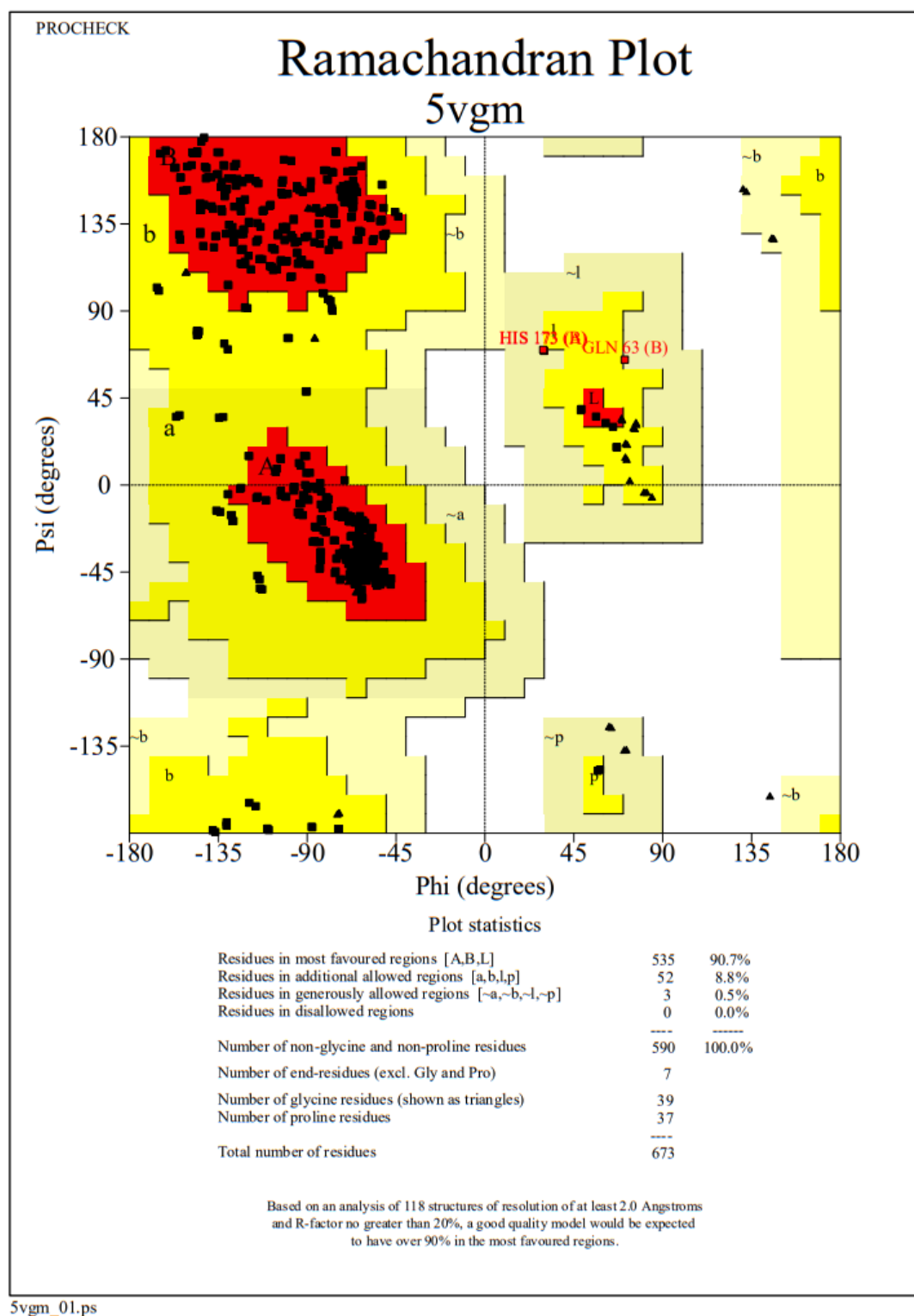

Figure S1. Main Ramachandran plot for 5VGM

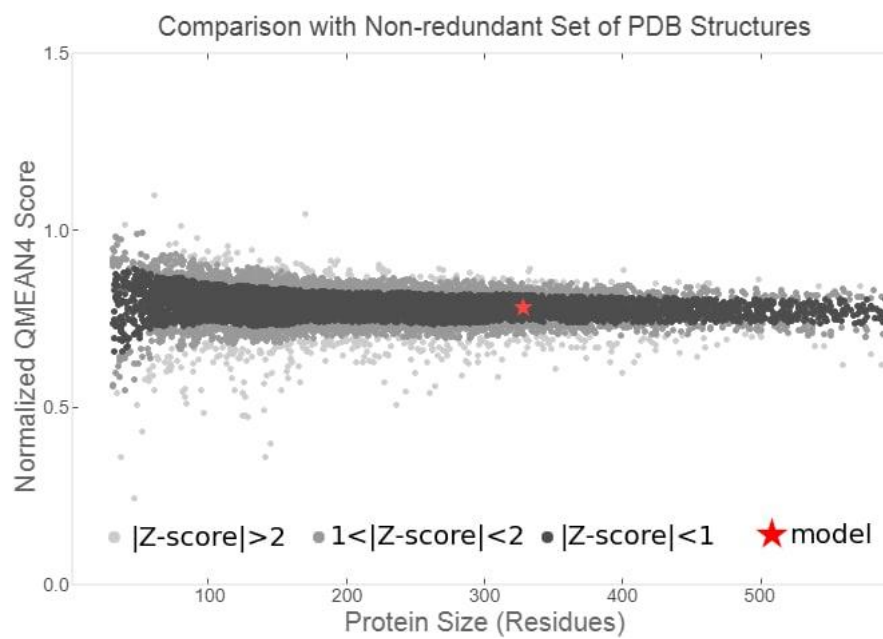

Figure S2. Quality -Comparison for 5VGM

Supplement: Supplementary file 1 [file molecules-28-01111-s001.zip › Supplement S1.pdf]
